# Lysophosphatidylcholine: A Novel Modulator of *Trypanosoma cruzi* Transmission

**DOI:** 10.1155/2012/625838

**Published:** 2011-11-01

**Authors:** Mário A. C. Silva-Neto, Alan B. Carneiro, Livia Silva-Cardoso, Georgia C. Atella

**Affiliations:** ^1^Instituto de Bioquímica Médica at Universidade Federal do Rio de Janeiro (UFRJ), 21940-590 Rio de Janeiro, RJ, Brazil; ^2^Instituto Nacional de Ciência e Tecnologia em Entomologia Molecular (INCT), 21940-902 Rio de janeiro, RJ, Brazil

## Abstract

Lysophosphatidylcholine is a bioactive lipid that regulates a large number of cellular processes and is especially present during the deposition and infiltration of inflammatory cells and deposition of atheromatous plaque. Such molecule is also present in saliva and feces of the hematophagous organism *Rhodnius prolixus*, a triatominae bug vector of Chagas disease. We have recently demonstrated that LPC is a modulator of *Trypanosoma cruzi* transmission. It acts as a powerful chemoattractant for inflammatory cells at the site of the insect bite, which will provide a concentrated population of cells available for parasite infection. Also, LPC increases macrophage intracellular calcium concentrations that ultimately enhance parasite invasion. Finally, LPC inhibits NO production by macrophages stimulated by live *T. cruzi*, and thus interferes with the immune system of the vertebrate host. In the present paper, we discuss the main signaling mechanisms that are likely used by such molecule and their eventual use as targets to block parasite transmission and the pathogenesis of Chagas disease.

## 1. Immune Response to *Trypanosoma cruzi* Infection in the Vertebrate Host

*T. cruzi* infects the vertebrate host through bite wounds produced in skin by a feeding bug or through the interaction of the parasite with conjunctival mucosa. Such interaction sometimes produces visible signs called Romaña's sign or chagoma inoculation. The histology of this initial site of infection is defined by an elevated number of mononuclear cells [1]. This first sign of infection suggests that *T. cruzi* can stimulate skin cells to produce mediators that trigger a local inflammatory response. Despite controversies about the mechanism of the pathogenesis of Chagas disease [2–5], until recently, some authors believed that the disease was limited to an acute phase, followed by a chronic phase that was considered an autoimmune disease, where the parasites would be physically linked to sites of inflammation in the heart and esophagus [6–8]. However, nowadays, the disease is considered multifactorial, with multiple and continuous interactions between pathogen and host [9]. After the incubation period of 2 to 3 weeks, infection with *T. cruzi* is manifested by the presence of a large number of parasites in the blood and tissues. Acute infection is accompanied by an excessive activation of the immune system that includes the production of high levels of cytokines, intense activation of T and B cells, lymphadenopathy, splenomegaly, and intense inflammation associated with tissue infection niches. The acute phase induces the development of an effective acquired immunity leading to the control of parasitemia. The chronic phase is considered lifelong and is associated with only a few parasites in the host. The beginning of chronic infection with *T. cruzi* is asymptomatic in most patients. However, with the advance of the disease, clinical manifestations become variable, ranging from no symptoms to the involvement of cardiovascular and/or gastrointestinal symptoms [10, 11]. Before the acquired immunity is established, the innate immune system appears to be essential for at least two important aspects of Chagas disease: control of replication of the parasite in the host tissue and progress of the inflammatory reaction. The latter, in turn, has been considered to be the main cause of tissue damage and dysfunction of certain organs in the host [11]. Some studies in experimental models of infection of *T. cruzi* suggest that the potent immune response to Th-1 CD4 and CD8 cells, with the production of specific inflammatory cytokines, such as interferon gamma (IFN-*γ*), tumour necrosis factor (TNF-*α*), and interleukin 12 (IL-12), as well as the production of reactive nitrogen species such as nitric oxide (NO), plays an important role in the control of parasitemia during the initial stage of the disease [4, 10–13]. Moreover, cells of innate immunity, such as natural killer (NK) cells, dendritic cells, and macrophages, are also key elements in the initial control of parasite replication [10–13].

In recent years, research on Chagas disease has focused on the investigation of the role of pathogen-associated molecular patterns (PAMPs) of protozoa, which are the targets of innate immune receptors. Also, the problem of identifying relevant receptors in innate immunity-parasite interactions during the evolution of the disease in the host has been addressed by several laboratories. This strategy ultimately aims at the development of therapeutic interventions through the use of PAMPs derived from parasites. Glycosyl-phosphatidyl-inositol (GPI) is the name given to the first glycoconjugate in *T. brucei* that was identified with the function of anchoring proteins on the cell surface [14–17]. PAMPs widely studied in *T. cruzi* are, in fact, GPI anchors. All evolutive forms of this parasite express on their surface GPI-anchored glycoproteins [14–17]. Some studies have identified GPI anchors isolated from trypomastigote-derived mucin-like glycoproteins (GPI-mucins) of *T. cruzi* as the molecules primarily responsible for stimulating the host immune system [18, 19]. Thus, *T. cruzi* GPI-mucins are able to activate macrophages and stimulate the production of proinflammatory cytokines, chemokines, and NO [20–22]. Innate immune response to *T. cruzi* has been studied extensively and is based on the activation of signaling pathways triggered by Toll-like receptors (TLRs). TLRs are proteins that recognize conserved motifs associated with several different pathogens; they trigger intracellular signaling cascades that ultimately lead to a complex host immune response [11, 12]. There are 10 TLRs described in humans and 12 in mice [11, 12]. Generally, the stimulus induced by GPI molecules occurs during the early phase of infection, where macrophages respond to trypomastigotes in a TLR-dependent mechanism and ultimately induce the production of IL-12 and TNF-*α* and trigger the responses of CD4 and CD8 cells through the production of IFN-*γ* [23]. Thus, macrophages activated by TNF-*α* and IFN-*γ* seem to have an important role in controlling parasite growth. Free GPI anchors or glycoinositolphospholipids (GIPLs) are also able to stimulate the host immune system. GIPLs are similar to GPIs but contain instead ceramide in their lipid moiety [18, 19]. 

TLRs 2, 4, and 9 are the major TLRs involved in innate immune response to *T. cruzi *[18, 24–29]. TLR2 has been identified as the main receptor responsible for macrophage activation by GPI mucins [18, 24–29]. According to Ropert and Gazzinelli [27], the receptor heterodimer composed of TLR2 and TRL6 is activated by GPI mucin and the CD14 coreceptor. Oliveira et al. [25] observed that GIPL from *T. cruzi* confers an inflammatory response via TLR4, promoting the recruitment of neutrophils into the peritoneal cavity of mice. Later, Medeiros et al. [26] demonstrated that this effect was partially dependent on the production of IL-1*β*. The genomic DNA of *T. cruzi* also plays an important role in proinflammatory response of the vertebrate host during infection, since TLR 9 is activated by CpG motifs from nonmethylated DNA [28, 29]. Besides the innate immune response mediated by TLRs, *T. cruzi* can also stimulate TLR-independent pathways that lead to the production of IFN-*β* and IFN-*γ*. In this case, this occurs due to a surge in intracellular calcium concentration which ultimately leads to the activation of calcineurin and calmodulin [30–32].

## 2. Lysophosphatidylcholine and Modulation of NO Production and Host Immunity

Lysophospholipids such as lysophosphatidylcholine (LPC), sphyngosylphosphoryilcholine (SPC), lysophosphatidic acid (LPA), and sphingosine-1-phosphate (S1P) regulate a large number of cellular processes. LPC is a derivative of phosphatidylcholine (PC) that arises by the loss of a fatty acid through the action of a phospholipase A_2_ (PLA_2_) or by transferring it to cholesterol by the action of a cholesterol-acetyltransferase [33]. LPC is involved in several physiological events and is already known as a central molecule in several pathological states, but it is especially present during the deposition and infiltration of inflammatory cells and deposition of atheromatous plaque [34–36]. Research directed towards LPC has increased greatly since the finding that these molecules are involved in atherosclerosis [37]. The idea that various phospholipases secreted by circulating leukocytes participate in this pathology was soon proposed. Thus, the current model suggests that diabetes and hypercholesterolemia contribute to generate a large number of LDL particles in plasma that can undergo oxidation of unsaturated fatty acids, generating an oxidized particle (oxLDL). Since on average 50% of LDL fatty acids are arachidonic acid and linoleic acid, the chances of such an oxidative event are huge. The oxLDL is a potential cause of the increased expression of inflammatory markers such as TNF-*α*, MCP-1, and MCSF that will attract differentiating monocytes to the lesion site. In this sense, LPC is one of the most powerful chemotactic signals for macrophages and is also generated by cells in the apoptotic process as mentioned above. OxLDL particles are recognized by various secretory PLA_2_ in the plasma, including type IIA, V, and X. Our group showed for the first time the presence of phospholipids and lysophospholipids in saliva and feces of the hematophagous organism *Rhodnius prolixus,* a triatominae bug vector of Chagas disease [38]. The major lipids present in *R. prolixus* saliva are PC and LPC [38]. Salivary LPC is an additional antihemostatic molecule that is part of the pharmacological arsenal injected into the bite site to allow the insect to feed. It inhibits platelet aggregation and increases the production of NO in endothelial cells. Thus, LPC was initially described as a molecule with antiplatelet and vasodilatory activities, and a few years later, its effect as an immunomodulator of *T.cruzi* infection was demonstrated [38, 39].

**Figure 1:**
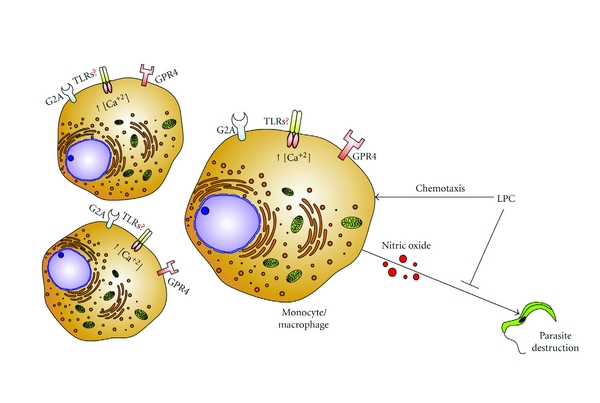
LPC-induced signaling on monocytes and macrophages. LPC is a signaling molecule that may act through different receptors on cell surface such as G2A and GPC4. Despite its description as a proinflammatory molecule, LPC-mediated signaling through TLRs is not demonstrated yet. LPC induces cell chemotaxis which ultimately increases the number of cells in the wound site. Also, LPC-treated cells undergo a decrease on NO synthesis when stimulated by parasite or LPS. Finally, a transient increase on intracellular calcium is also reported in such cells. These combined effects enhance the number of cells prone to *T. cruzi *invasion.

The role of LPC as a modulator of *T. cruzi* transmission occurs by three main mechanisms summarized on Figure 1 and mentioned as follows.

 (1) LPC is a vector-derived molecule. It acts as a powerful chemoattractant for inflammatory cells at the site of the insect bite. This event will provide a concentrated population of cells available for *T. cruzi* infection [38, 39].

(2) LPC increases macrophage intracellular calcium concentrations that ultimately enhance parasite invasion.

(3) LPC inhibits NO production by macrophages stimulated by either live *T. cruzi*, LPS, or LPS in the presence of IFN-*γ*, and thus interferes with the immune system of the vertebrate host [39]. 

The above findings demonstrate that LPC is now a signaling molecule with effects beyond that of counteracting host blood hemostasis, since it acts as modulator of NO biology and parasite transmission [40–43]. Macrophages are intimately related to the establishment of acute infection with *T. cruzi*, since the success of the infection depends on the initial invasion of these cells [44–46]. This leads to the assumption that salivary LPC may facilitate the parasite infection, favoring not only insect feeding, but also preparing the environment for the arrival of the parasite, minutes or hours after the initial bug bite. Recent results obtained by our group demonstrated that injection of salivary LPC into host skin followed by parasite inoculation in the same site minutes later ultimately increases blood parasitemia from 3- to 6-fold in animals infected with *T. cruzi. *LPC's effect on parasitemia is mainly achieved by the activation of macrophage chemotaxis and immunosuppression of NO production induced by the parasite. We also showed an increase in the rate of association of the parasite with macrophages induced either by 500-fold diluted saliva or by LPC. This was the first demonstration of a potentiating factor of transmission of Chagas disease and the first implication of a lysophospholipid in an infectious disease [39].

 The activation of receptors that recognize the parasite by the presence of specific structures on its surface stimulates host cells to produce TNF-*α*, IL-12, and NO, as mentioned above. Depending on the MyD88 adapter protein, TLRs 2, 4, and 9 have been implicated in the network used by the immune system of the mammalian host to control infection by *T. cruzi* [14–18]. Campos et al. [18, 23] were the first to demonstrate the involvement of TLR2 in the interaction between the parasite and host macrophages. The expression of TLR2 is essential for the induction of IL-12, TNF-*α*, and NO, and this receptor is activated by parasite-derived molecules such as GPI anchors, which have been isolated from the surface of trypomastigotes of *T. cruzi* [14–18]. The production of NO but not IL-12 by *T. cruzi*-exposed macrophages is not affected by bug saliva [39]. Curiously, in bone-marrow-derived macrophages obtained from TLR2-deficient mice, the production of IL-12 is largely suppressed by LPC. These data indicate that in some cell types, the production of this cytokine may be affected by this lysophospholipid through a TLR2-independent mechanism. 

Moreover, GIPLs from *T. cruzi* are TLR4 agonists with proinflammatory effects [25, 26]. We showed that NO production, induced by the parasite or by lipopolysaccharide (LPS), another ligand of TLR4, either in murine peritoneal macrophages or bone marrow-derived macrophages, is blocked in both cases by LPC even in the presence of IFN-*γ* *in vitro* [39]. The ability of LPC to reverse the induction of NO production in all cases, almost independently of the ligand type, suggests that this lysophospholipid must act by a unique pathway. In this regard, the receptors involved in cell signaling induced by LPC, in general, exhibit a certain promiscuity with respect to the ligand and vice versa. In the case of LPC, different receptors have been proposed for this molecule, including G2A, a G protein-coupled receptor, and GPR4, another important candidate [45, 47–49]. Despite the controversy generated in the literature due to the low reproducibility of the studies using radioactive LPC and its interaction with candidate receptors, the ability of G2A to bind fatty acids and protons is noteworthy [33]. Thus, G2A remains in the literature as the best-known receptor involved in the adaptation of the signal induced by LPC [47–49]. Moreover, the redistribution of G2A receptor itself and the exposure of TLR4 are influenced by LPC metabolism [33, 50]. In this case, the content of intracellular LPC is finely controlled by the activity of a lysophosphatidylcholine acyltransferase (LPCAT), an enzyme that uses LPC as a substrate and generates phospholipids as the product of its action. The treatment of monocytes with LPS activates this enzyme and increases the transport of TLR4 to membrane rafts in these cells [50]. Since the LPCAT inhibitor used, 5-hydroxyethyl 5′3′ thiophenol pyridine (HETP), increases the lysophospholipids/phospholipids ratios, it reverses the effect of LPS [50]. Thus, it seems appropriate to propose that in the presence of *T. cruzi*, one should conduct a map of the distribution of both receptors, G2A and TLR4, in the presence and in the absence of LPC using both proteomic and immunological methods [33, 50]. 

During programmed cell death, LPC is generated by a calcium-independent PLA_2_ activated by caspase-3. Thus, LPC acts as a chemotactic *find-me* signal that attracts the phagocyte to the apoptotic cells [49, 51, 52] and as an *eat-me* signal involving recruitment of complement proteins for recognition by phagocytes [49, 51, 52]. Such LPC-induced chemotaxis is very interesting, because in Chagas disease, the uptake of apoptotic cells by macrophages infected with *T. cruzi* stimulates parasite growth [53]. In addition, it has been shown that *T. cruzi* infective stages are able to generate lipid messengers, including LPC, that modulate host cell signaling [54]. Regarding adaptor molecules mobilized in response to LPC, it is known that in most cell types, there is the involvement of isoforms of protein kinase C [55]. Probably it is the type of isoform activated in each cell that directs the intensity and type of response triggered by LPC in that specific cell type. When combined with different types of TLRs and adapters, LPC-mediated signaling must produce a specific and still poorly understood repertoire of immunosuppression. 

## 3. Vector Phospholipases and Eventual Target to Block *T. cruzi* Transmission

PLA_2_ is an enzyme family present in various organisms such as viruses, bacteria, plants, and animals. According to studies done on mammals, the action of PLA_2_ is important for the remodeling of cell membranes, lipid digestion, cell signaling, and immune defense of the host as well as production of various lipid mediators [56–61]. In insects, the phospholipases that have been studied are related to the venom injected into their prey, the physiology of digestion, immunity, and reproduction [61]. Among the published studies on phospholipase activity in arthropods are those reporting the presence of such enzymes in the salivary glands of *Manduca sexta* [62] and in the saliva and salivary glands of *Amblyoma americanum* [63, 64]. These studies have found a correlation between PLA_2_ activity and digestion. In addition, Zhu et al. [64] suggested another role for this activity, linking it to the production of prostaglandins, promotion of vasodilation and the suppression of inflammation and immunity. The production of prostacyclin may also lead to the inhibition of platelet aggregation and the induction of vasodilation. Furthermore, platelet-activating factor (PAF) acetyl hydrolase, a member of the GVII family of PLA_2_ enzymes, is a serine-dependent hydrolase that does not require Ca^2+^ for activity. This enzyme cleaves the acetyl group from the *sn*-2 position of the phospholipid, and in the case of PAF, there is the hydrolysis of the *sn*-2 ester bond, releasing acetate and biologically inactive lyso-PAF [65, 66]. Cat flea (*Ctenocephalides felis*) salivary gland homogenate has a PAF-acetylhydrolase activity, and the estimated amount of activity in a single pair of salivary glands (~5 pmol/min) is of the right order of magnitude to induce a localized anti-inflammatory/allergic effect [67]. Extracellular PAF is proinflammatory and acts via very high affinity G-coupled protein receptors, causing activation of platelets, neutrophils, and monocytes [66]. The hypothesis is that PAF-acetylhydrolase activities from saliva can downregulate inflammatory and immune reactions mediated by PAF released from host cells. This may happen as a reaction to injected cat flea saliva and may be interrupted by host grooming or scratching the locale of the bite. Besides these reports, phospholipases also have been identified in transcriptomes of saliva or salivary glands of some hematophagous arthropods such as the soft ticks *Ornithodoros coriaceus* [67, 68] and *O*.* parkeri* [69], of hard ticks such as *Ixodes pacificus *[70], and in insects such as *Anopheles funestus* [71], *Phlebotomus arabicus* [72], and *Glossina morsitans* [73]. 

Zeidner et al. [74] have suggested that aside from facilitating some tick digestive processes, it is possible that secretion of PLA_2_ into the feed site creates some protective barrier against bacteria that can be carried into the wound. They demonstrated that the borreliacidal activity found in *A. americanum* saliva is most probably due to the enzymatic effects of PLA_2_ and that it would directly and rapidly kill *Borrelia burgdorgeri* through the digestion of membrane lipids, composed by a majority of PC and phosphatidylglycerol. The authors hypothesize that high level of PLA_2_ enzymatic activity present in saliva is related to *A. americanum's* refractoriness to *B. burgdorgeri.* Other studies have demonstrated the importance of PLA_2_ in the infection processas elicited by pathogens such as *Toxoplasma gondii*, *Cryptosporium parvum*, *Entamoeba histolytica*,* Leishmania amazonensis*, and *T. cruzi* [75–77]. Moreover, Connelly and Kierszenbaum [44] showed that the presence of PLA_2_ significantly increased the association between *T. cruzi* and macrophages, and they suggest that this effect relies on alterations of the parasite membrane, since it was induced by pretreatment of parasite membranes with PLA_2_ but not macrophages. But nowadays, as cited above, the presence of PLA_2_ in salivary secretions of *T. cruzi* vectors implies LPC generation and its further involvement in the inflammatory process that occurs during infection. 

PLA_2_ enzymes from snake venom induce a wide spectrum of pharmacological effects, including anticoagulant proprieties that can be mediated by hydrolysis of phospholipid or by a nonenzymatic mechanism, such as when PLA_2_ from* Naja nigricollis *venom binds factor Xa in the coagulation cascade through the specific anticoagulant site on its surface [78]. Our group is investigating a further role for the LPC present in the saliva of vectors, which we believe is related to muscular paralysis. Rigoni et al. [79] have shown that lysophospholipids, in particular LPC, can block the exocytosis of neurotransmitters, thus paralyzing the muscle. In this context, using the predator insect *Belostoma anurum* as model, we showed that the salivary LPC also has this property. Our hypothesis is that *B. anurum* uses lysophospholipids as a way to paralyze the prey while it feeds, since it makes an extraoral digestion [80]. We obtained similar results with LPC from *R. prolixus*, with less pronounced blockage of exocytosis. LPC action may be more local in order to avoid disturbing the host. Thus, the above data show that the presence of LPC generated by PLA_2_s in salivary secretions of predators and blood-sucking arthopods is widespread in the animal kingdom, and this molecule may be a surviving trace of ancient feeding habits. 

Another aspect that should be emphasized is that PLA_2_s also generate free fatty acids that can be converted to eicosanoids. Eicosanoids are polyunsaturated fatty acids of 20 carbons that act as local mediators of short half-life; they are derived from arachidonic acid (20 : 4 n-6) or other polyunsaturated 20-carbon precursors (20 : 3 n-6 and 20 : 5 n-3). Arachidonic acid is esterified in phospholipids of plasma membranes, these being released by the action of PLA_2_. The biological action of arachidonic acid products requires its oxygenation, which can take place in three different ways: (a) via the cyclooxygenases that generate prostaglandins and thromboxanes, (b) via the lipooxygenases that generate leukotrienes and lipoxins, and (c) via the cytochrome P-450, which generates epoxides [81]. Physiological processes that usually involve autacoids, hormones, and growth factors may stimulate the release of arachidonic acid, as already widely described in mammals, as in mediating immune and inflammatory response of late vertebrates [82]. Recently, the involvement of thromboxane A_2_ (TXA_2_) in the process of vertebrate host infection by *T. cruzi* [83] was demonstrated. The same group showed that the eicosanoid TXA_2_ is prevalent in all life stages of the parasite. Thus, in infected mice, the parasite itself may account for 90% of the total TXA_2_ in plasma. In this regard, it is noteworthy that the production of TXA_2_ from arachidonic acid occurs by the cyclooxygenase pathway. Accordingly, results from our laboratory indicate that half of the fatty acids ingested along with blood are unsaturated and about half of them are arachidonic acid. So, if a pool of TXA_2_ is a prerequisite for the process of infection of host cells by any pathogen, that pool could be generated during the final stages of blood digestion in the vector at the expense of fatty acids released there. A triacylglycerol-lipase activity was identified in the gut lumen of blood-fed insects and is probably involved in the digestion of lipids from the blood meal. These lipase activities and also the metabolism and fate of lipids that are generated during digestion of ingested blood were studied and characterized [84]. However, neither the dynamic generation of free fatty acids in insects infected with *T. cruzi* nor their processing to TXA_2_ in the final stage of the digestive process has ever been assessed in any vector. Thus, an attractive model for the future might involve the silencing of a PLA_2_ gene in the saliva of Chagas disease vectors to obtain LPC-depleted or LPC-free saliva. The saliva of these insects would be expected to lower the rate of infection of the vertebrate host.

## 4. The Role of Host Plasma LPC in *T. cruzi* Infection

The original studies that implicated LPC in the pathogenesis of atherosclerosis tended to highlight the presence of this phospholipid in atheromatous lesions [37]. However, the origin and dynamics of the formation of this molecule remained unknown for many years. Wilensky et al. [85] identified the main enzyme responsible for the generation of LPC, Lp-PLA_2_, also known as PAF-acetyl hydrolase or phospholipase VIIA, which is secreted by leukocytes and associated with plasma lipoproteins, especially LDL. This enzyme recognizes and cleaves oxLDL and oxidized phospholipids, generating LPC and free fatty acid oxidation (oxNEFAS, or oxidized nonesterified fatty acids). The LPC, as previously mentioned, is a potent proinflammatory molecule capable of leukocyte recruitment and activation with induction of apoptosis. Demonstration of Lp-PLA_2_ in the necrotic core of atheromatous lesions and fibrous cap of vulnerable plaques supports our current views of the importance of this enzyme in atherogenesis. An important therapeutic option is the selective inhibition of Lp-PLA_2_. Wilensky et al. [85] showed that the administration of darapladib (GlaxoSmithKline) in experimental models selectively reduces the activity of this enzyme, attenuates the formation of LPC, and reduces the formation of atherosclerotic plaques with negative regulation of proinflammatory genes in macrophages and T lymphocytes. Chagas disease treatment aims to slow the progression of myocardial impairment caused by invasion of the patient's heart by the parasite. Some of the drugs used to treat Chagas disease cause changes in the patients plasma lipid profile, leading to high concentrations of LDL. During treatment, these patients are likely to present favorable conditions for LDL oxidation and generation of LPC, which will certainly trigger the proinflammatory phenotype, thereby maintaining levels of reinfection of myocardial cells. In this sense, it would be important to evaluate the generation of LPC in Chagas disease patients treated with various categories of drugs in order to verify the formation of this lipid mediator. Likewise, chronic treatment with the drug in healthy experimental animals should be carried out to identify any effects on the levels of LPC and subsequent susceptibility to infection by *T. cruzi*. In conclusion, darapladib may constitute a novel tool with dual use: to optimize the current therapeutic treatment of chronic chagasic patients and to experimentally modify the plasma levels of LPC in mice to determine whether the reduction of such levels decreases the susceptibly to infection by *T. cruzi.*
